# Surgical Fixation of Three- and Four-Part Proximal Humeral Fractures Using the Proximal Humeral Interlocking System Plate

**DOI:** 10.7759/cureus.25348

**Published:** 2022-05-26

**Authors:** Ahmed Y Saber, Umar N Said, Abdelmonem H Abdelmonem, Hassan Elsayed, Mohamed Taha, Walid Hussein, Khalid Al-Hashimi, Omar El-Omar, Mohamed Elbeshbeshy

**Affiliations:** 1 Trauma and Orthopaedics, Calderdale and Huddersfield National Health Service (NHS) Foundation Trust, Huddersfield, GBR; 2 Trauma and Orthopaedics, Huddersfield Royal Infirmary, Huddersfield, GBR; 3 Trauma and Orthopaedics, West Hertfordshire Hospitals National Health Service (NHS) Trust, Watford, GBR; 4 Trauma and Orthopaedics, Rotherham National Health Service (NHS) Foundation Trust, Rotherham, GBR; 5 Trauma and Orthopaedics, Birmingham Children's Hospital, Birmingham, GBR; 6 Vascular Surgery, Royal Shrewsbury Hospital, Shrewsbury, GBR; 7 Trauma and Orthopaedics, King's Mill Hospital, Nottinghamshire, GBR

**Keywords:** fracture stabilization, internal fixation, locking plate, humerus, fracture

## Abstract

Introduction

The management of proximal humeral fractures ranges greatly from conservative management to surgical treatment. For those fractures requiring surgical treatment, internal fixation is the primary method. The aim of internal fixation is to achieve rigid fracture fixation until union occurs, return of shoulder range of motion, and minimise intra-and postoperative complications. The aim of this study was to evaluate the results of the Proximal Humeral Interlocking System Plate (PHILOS) used for the treatment of three-and four-part proximal humeral fractures.

Materials and methods

This study included 30 patients with a mean age of 54 years (range 20-80 years). Results were checked post-operatively with standard radiographs and clinical evaluation according to the Constant-Murley shoulder score. All patients were followed up for 12 months.

Results

Union was achieved in all patients with a mean neck/shaft angle of 130° (range 108°-150°). The mean Constant-Murley score at the final follow-up was 82.28 (range 67-96) correlating with good results. No patients developed an intraoperative or postoperative vascular injury, wound complications, or avascular necrosis of the humeral head.

Conclusion

Our study has shown that the surgical treatment of three- and four-part proximal humeral fractures with the use of the PHILOS plate leads to a good functional outcome. It has also demonstrated the PHILOS plate and is an effective system for fracture stabilisation provided the correct surgical technique is used with awareness of potential hardware complications.

## Introduction

Proximal humeral fractures account for around 5-6% of all adult fractures, however, they are more common in patients over 65 years of age [1]⁠. Proximal humeral fractures in the elderly usually occur in a low energy setting such as a fall from a standing height and are associated with osteoporotic age-related degeneration in their bone density [1]. Proximal humeral fractures in younger patients are associated with higher energy mechanisms of injury such as road traffic accidents [2].

The most widely used classification for proximal humeral fractures was devised by Charles Neer whereby the proximal humerus is divided into four parts. These four parts are the humeral head, the greater tuberosity, the lesser tuberosity, and the humeral shaft. Displacement is determined per part and the criteria for displacement include whether or not the fractured part is angulated by more than 45 degrees, or if the fracture is displaced by more than 1 cm [3]⁠.

Non and minimally displaced fractures tend to be stable and as such are treated non operatively. Three- and four-part displaced fractures are unstable and also carry a higher likelihood of complications, most notably disruption of the proximal humeral blood supply [4]⁠. In contrast to non and minimally displaced fractures, the management of displaced or unstable fractures can be controversial. Internal fixation can lead to unpredictable outcomes in patients with proximal humeral fractures, particularly in those with osteoporosis or highly comminuted fractures. Different treatment modalities for these types of fracture include the usage of non-contoured proximal humeral plates, intramedullary nailing, and hemiarthroplasty. These modalities are associated with a variety of complications including implant failure, non or malunion, and osteonecrosis of the humeral head [5]⁠.

The Proximal Humeral Interlocking System Plate (PHILOS) was developed in an attempt to reduce complications associated with other fixation methods. The plate is contoured to the shape of the proximal humerus and is versatile due to being able to accept both standard and locking screws. The plate does not require any compression which reduces the risk of reduction being lost and helps to preserve blood supply to the proximal humeral head. The utilisation of locking screws in conjunction with the PHILOS plate improves axial stability and further decreases the risk of reduction being lost [5]⁠. Data has also shown that fixation with anatomical plates such as the PHILOS plate in the osteoporotic bone can resist physiological load to a higher degree than angular stable plates or intramedullary nails [6]⁠.

This prospective study was undertaken to evaluate how effective the PHILOS plate was in the surgical fixation of three- and four-part proximal humeral fractures.

## Materials and methods

This study included 30 patients who presented to Al Razy Hospital, Kuwait. Inclusion criteria for this study were displaced three- and four-part fractures of the proximal humerus that were diagnosed clinically, and via AP and lateral radiographs. Exclusion criteria included a previous fracture to the proximal humerus, or patients presenting more than three weeks after the injury. Computed tomography (CT) scans were done for selected patients in order to determine any intra-articular extension of the fracture. All patients were treated using the PHILOS plate

Our cohort consisted of 18 men and 12 women with a mean age of 54 years (range 20-80 years). The injuries were right-sided in 19 cases and left-sided in the remaining 11 patients. In 14 patients the mechanism of injury was a road traffic collision whereas in the remaining 16 patients the mechanism was direct trauma to the shoulder via a fall. Twenty patients had a three-part proximal humeral fracture of which five were associated with a shoulder dislocation, and 10 patients had a four-part proximal humeral fracture of which three were associated with a shoulder dislocation.

Sixteen patients had isolated three- or four-part proximal humeral fractures, whereas the remaining 14 also had additional injuries including fractures of the humeral shaft, femur, tibia, distal radius, and contralateral humerus. None of the patients in our study had open fractures. All patients on admission were examined for evidence of neurovascular injury, one patient had pre-operative radial nerve injury and one patient had a complete brachial plexus injury

**Figure 1:**
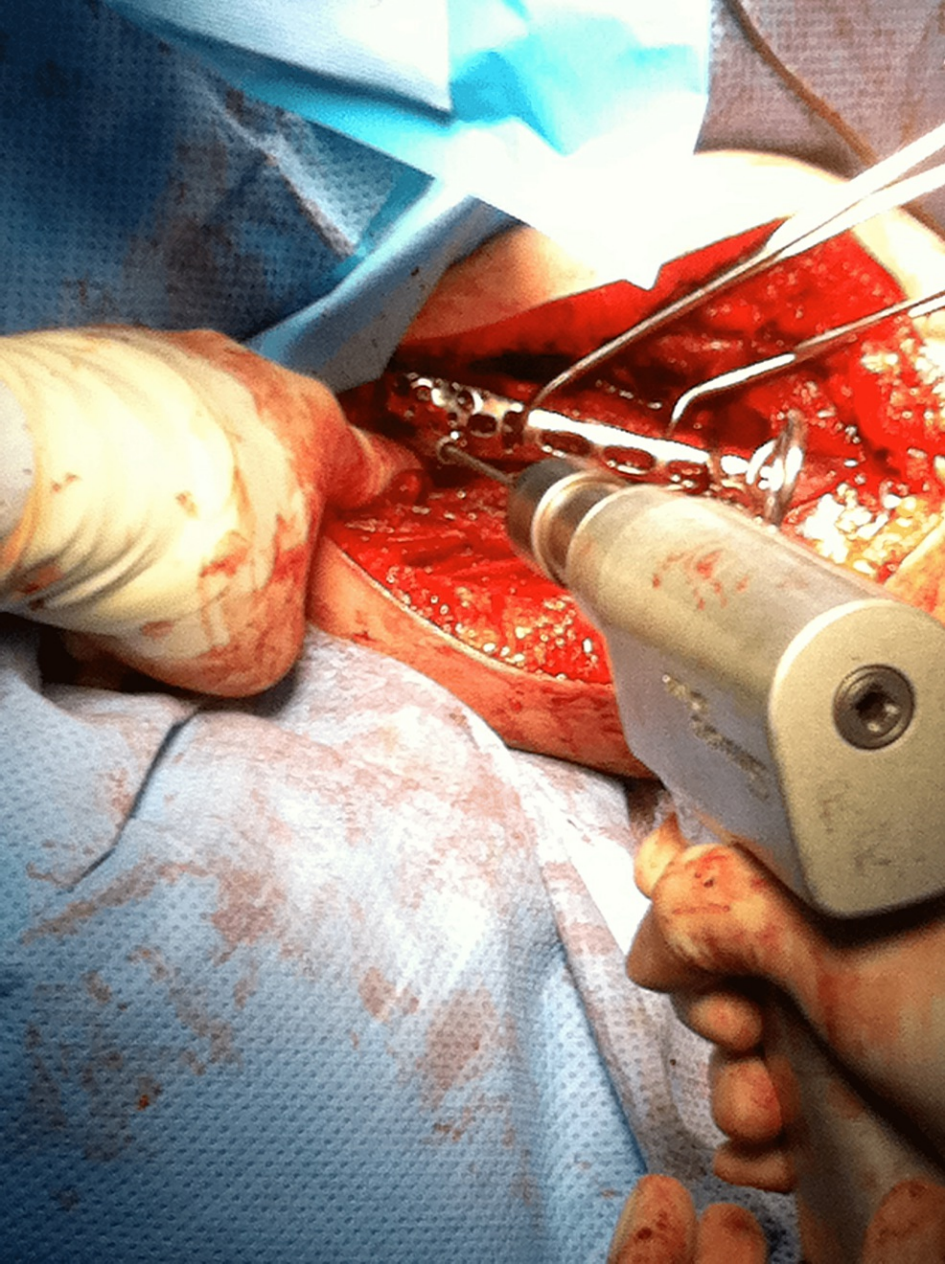
Intra-operative picture showing placement of the PHILOS plate PHILOS: Proximal Humeral Interlocking System

The surgery in all patients was performed under general anaesthetic with the patient in the beach chair position. The proximal humerus was accessed using a delto-pectoral approach. Humeral head and tuberosity fragments were manipulated and temporarily fixed using K-wires. The PHILOS plate (DePuy Synthes, Massachusetts, USA) was placed 8-10 mm distal to the rotator cuff attachment on the upper edge of the greater tuberosity as seen in Figure 1. Care was taken to avoid placement of the plate too high due to the increased risk of subacromial impingement, and also to ensure that there was adequate space between the plate and the long biceps tendon.

**Figure 2:**
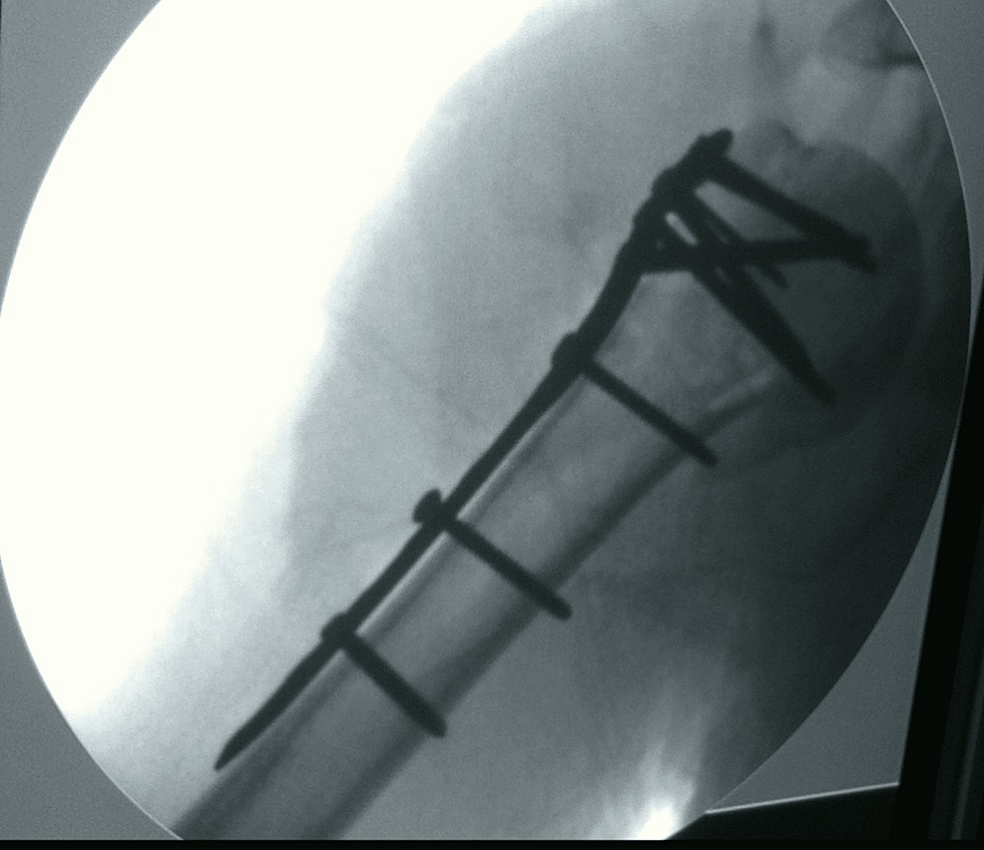
Intra-operative radiograph showing AP view of applied PHILOS plate AP view: Anteroposterior view; PHILOS: Proximal Humeral Interlocking System

**Figure 3:**
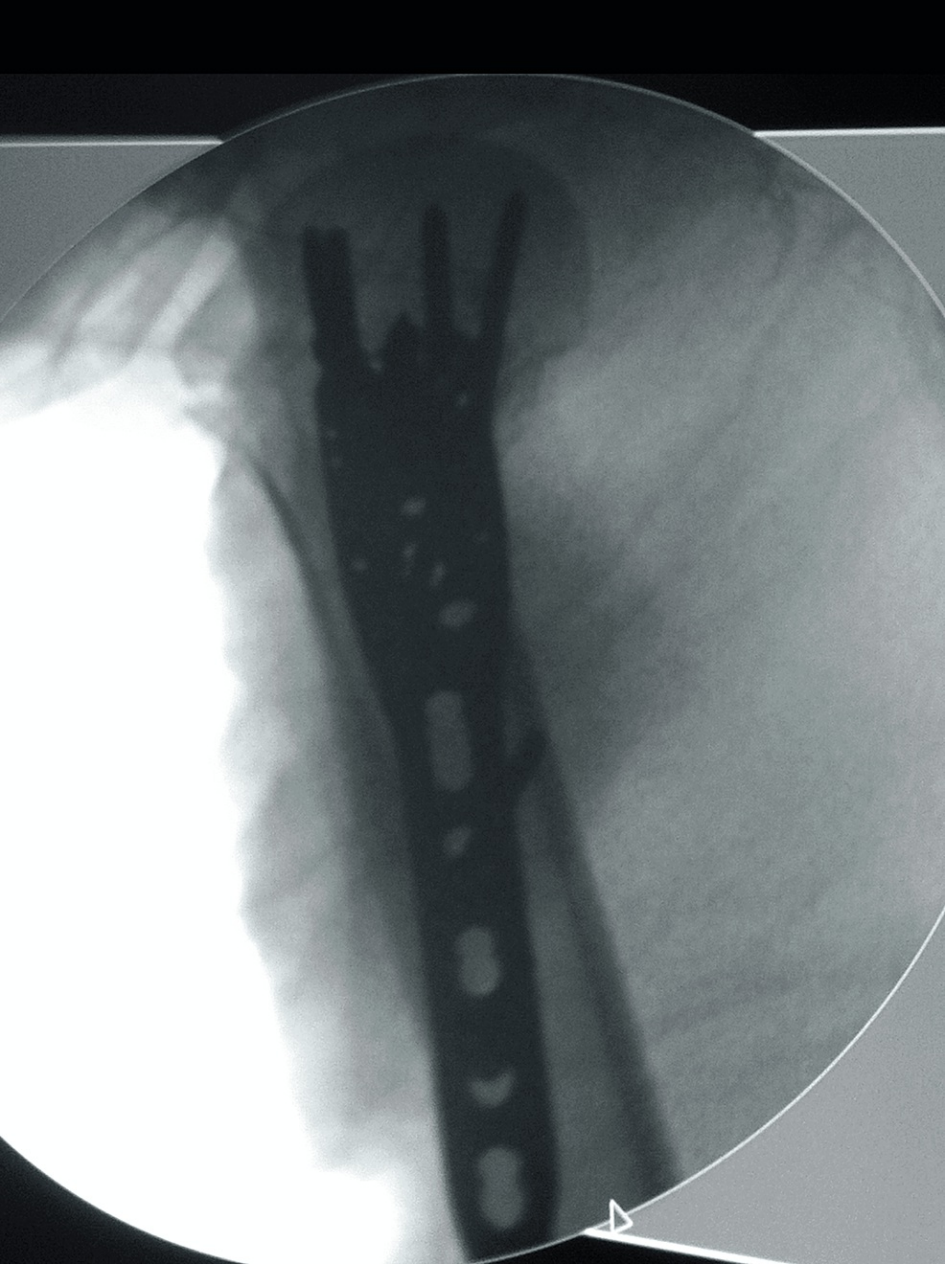
Intra-operative radiograph showing lateral view of applied PHILOS plate PHILOS: Proximal Humeral Interlocking System

A drill-sleeve system was attached to the aiming device and 2 K-wires were inserted for provisional fixation. The plate was fixed distally first as the usage of compression screws here generated compression between fragments prior to insertion of locking screws. The plate was then secured proximally with atleast four locking screws, more were used in cases of poor bone quality. Intra-operative images of this can be seen in Figures 2-3.

Postoperatively passive mobilization was started immediately. Active mobilization without the addition of weight was initiated the fourth week after surgery, with full active mobilization initiated in week six. Progressive strengthening exercises of the shoulder were commenced at week twelve. All patients were followed up with standard radiographs and clinical evaluation according to the Constant-Murley shoulder score at 1, 3, 6 and 12 months post surgery.

## Results

**Figure 4:**
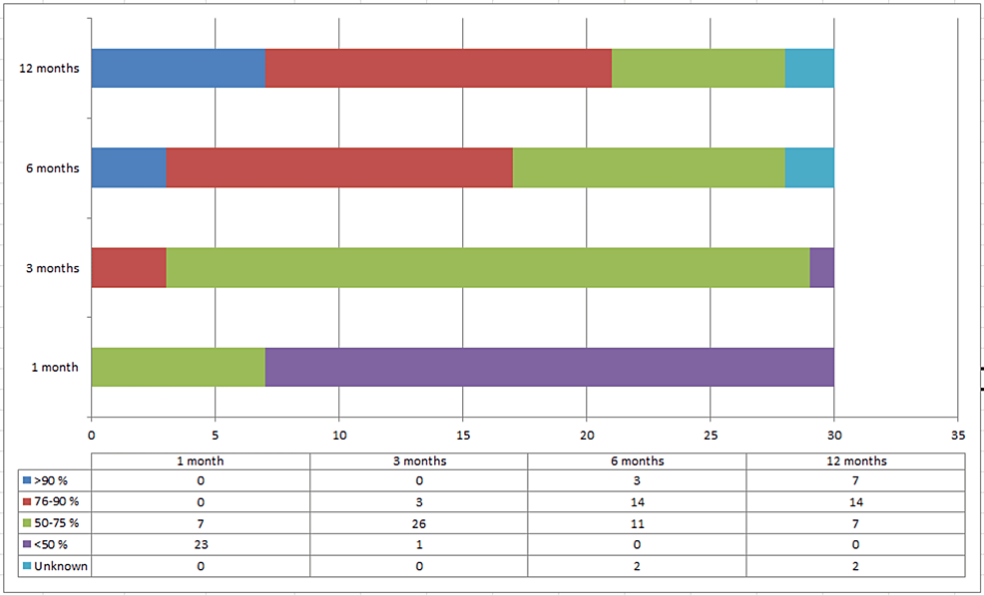
Comparison between Constant scores at 1, 3, 6 and 12 months post-operatively.

At 12 months the mean Constant score of the cohort was 82.28 (range 67-96). The mean Constant score improved significantly between each follow-up interval (p < 0.05) as seen in Figure 4. The mean Constant scores at 1, 3, 6, and 12 months can be seen in Table 1 below.

**Table 1:** Mean Constant scores at 1, 3, 6 and 12 months post-operatively.

Follow up period	Mean Constant score
1 month	43.37
3 months	63.16
6 months	77.57
12 months	82.28

Our results also demonstrated that patients aged 40 years or less had a higher mean Constant score at 12 months of 94.11 compared to those aged over 40 of 80.22 (p < 0.05). The mean Constant score in patients with three-part fractures was significantly higher (p < 0.05) at 84.70 whereas in patients with four-part fractures the score was 76.25. There was no significant difference in patients who had a dislocation associated with the proximal humeral fracture. Those sustaining a dislocation had a mean Constant score of 82.20 compared to 82.42 in those who did not sustain a dislocation.

The mean neck-shaft angle postoperatively was 133° (range 108° - 150°). No patients in the study developed intraoperative or postoperative vascular injuries, would complications, infections, or avascular necrosis of the humeral head. Three patients suffered from nerve injuries. Two of them had radial nerve palsy, one pre-operatively and one post-operatively, both showed complete recovery after six months. The third patient suffered from complete brachial plexus injury in association with the fracture.

Two patients suffered from mal-union. Intra-articular screw perforation was noticed in four patients, however, none of them required further surgical intervention. One patient suffered from shoulder stiffness and limitation of shoulder movement for three months post-operatively which improved by 12 months.

## Discussion

The incidence of proximal humeral fractures is on the rise due to an increase in life expectancy and the resultant elderly population. Some studies have estimated that the number of proximal humeral fractures may triple by the year 2030 [7]⁠. The majority of these fractures in the elderly are related to osteoporosis and low-velocity injuries. Conversely, fractures in the younger population tend to be caused by high-velocity and are much more complex with greater comminution and potential for soft tissue injury [8]⁠.

Traditional methods of internal fixation of proximal humeral fractures can be divided into semi-rigid (K wires, screw fixation, tension band wiring) or rigid (conventional plating or intramedullary nailing [9]⁠). Internal fixation using conventional non-locking plates has been shown in some studies to be the strongest method of fixation, but this is only the case in healthy non-osteoporotic bone. The usage of these conventional plates in osteoporotic bone leads to a high failure rate, particularly in individuals with three- and four-part fractures [10]⁠.

The PHILOS plate was developed as a next-generation plate to help avoid complications associated with conventional plates. The PHILOS plate is biomechanically superior to conventional plates due to many different factors. Some of these include high resistance to avulsion in osteoporotic bone, the combined ability to use fixed and locking screws, three-dimensional placement of screws into the humeral head, and early mobility due to the construct being immediately rigid and stable [7]⁠. The PHILOS plate also preserves vascularization due to reduced compression of the periosteum [11]⁠. The absence of any case of avascular necrosis of the humeral head in our study supports this.

In this prospective study, 30 patients were treated who sustained a three- or four-part proximal humeral fracture and were followed up until 12 months. The mean age of patients was 54.0 years (range 20-80 years). The mean constant score of our study was 82.28 (range 67-96). The number of patients included in our study is comparable to those seen in the literature for similar studies seen in Table 2.

**Table 2:** Comparison of our study with other studies. Iacobellis et al. [12]⁠; Johannes et al. [13]⁠; Charalambous et al. [14]⁠; Sharafeldin et al. [15]⁠; Rosario et al. [16]⁠; Soliman et al. [17]⁠

	Our study	Iacobellis et al.	Johannes et al.⁠	Charalambous et al.⁠	Sharafeldin et al.⁠	Rosario et al.⁠	Soliman et al.
Number of patients (n)	30	30	50	23	27	32	39 (only 27 treated with proximal humeral plate)
Mean age (years)	54	64.3	70	63	61.1	52	29.6
Female % Male %	40% 60%	70% 30%	60% 40%	47.8% 52.2%	66.6% 33.4%	62.5% 37.5%	25.64% 74.36%
Type of fracture	3 and 4 part	3 and 4 part	2, 3, and 4 part	2, 3 ,and 4 part	2, 3, and 4 part	2, 3, and 4 part	4 part fractures in young adults
Follow up period (months)	12 months	21 months	24 months	6 months	12 months	6-24 months	26 months
Mean neck-shaft angle (°)	130°	133.7°	-	127.2°	-	-	-
Mean constant score	82.28	68.6	76	-	64	79	77

The functional outcome following surgery was measured using the Constant-Murley shoulder outcome score. This is a tool that consists of four parameters to assess shoulder function, which include pain, range of motion, strength, and daily activity (sleep, work, recreation/sport). The minimum score is 0 with the maximum being 100 [18]⁠. From the analysis of the literature, our mean Constant score of 82.28 at 12 months is considered acceptable.

Patients with a three-part fracture had an average constant score of 84.70 whereas those with a four-part fracture had an average constant score of 76.25 (p < 0.05) which was statistically significant. There was no significant difference in outcome for those patients who suffered an associated dislocation compared to those who did not.

The complication rate in our study of 33.3% is comparable to the literature where complication rates are reported to be between 32-50% [19]⁠. Table 3 summarizes the complications that occurred in this study.

**Table 3:** Table to show complications seen in our study.

Complication	Number of cases	Percentage of total cases in study (30)
Intra-articular screw perforation	4	13.3%
Nerve injuries	3	10%
Mal-union	2	6.6%
Shoulder stiffness	1	3.3%
Total	10	33.3%

Intra-articular screw perforation was the most frequent complication seen in four patients (cases no 3, 6, 7, and 12). Two of these patients were scheduled for screw removal, one patient did not continue to follow up and the final patient had minimal perforation and did not require surgical intervention. It is possible that the cause of screw protrusion could have been either due to inadequate intra-operative screening or early post-operative collapse of the fracture. These results are similar to the study by Charalambous et al. where four of 25 cases (16%) also suffered from intra-articular screw protrusion [14]⁠.

Three patients suffered from nerve injuries, two of these had radial nerve palsy, one case pre-operatively (case no 4) and the other post-operatively (case no 18). Case no 4 and 18 showed complete recovery after six months following physiotherapy. Unfortunately, the third patient (case no 12) suffered from a complete brachial plexus injury in association with the fracture and did not regain function.

Two patients suffered from mal-union, case no 16 suffered from mal-union with screw loosening but refused further surgical intervention. The other patient (case no 2) demonstrated varus mal-union with a neck-shaft angle of 108°. This patient's progress was still good with a final Constant score at 12 months of 73.0. A study by Soliman et al. demonstrated 1 of 39 cases demonstrating mal-union (2.6%) [17]⁠.

One patient suffered from shoulder stiffness and limitation of shoulder movement that resolved three months post-operatively with physiotherapy (case no 10). No patient developed wound dehiscence or infection. Avascular necrosis of the humeral head is a well-recognized complication with research by Thanasas et al. reporting an incidence of 7.9% in proximal humeral fractures treated with locking plates [20]⁠. No patients developed avascular necrosis of the humeral head in our study suggesting good operative technique and the PHILOS plate being a superior method of fixation.

Analysis of the mean neck-shaft angle revealed a mean value of 130° (range 108°-150°). Twenty-seven cases had a value between 120°-145° which is considered to be within normal limits. In three patients (cases no 1, 2, and 3), case number 1 had an angle of 148° and a Constant score of 73.0, case number 2 had an angle of 108° and a constant score of 73.0, case number 3 had an angle of 150° and a constant score of 92.0. These results are consistent with work carried out by Iacobellis et al. with a mean angle of 133.7° and Charalambous et al. with a mean angle of 127.2° [12,14]⁠.

A limitation in our study is the lack of a comparative group, therefore we cannot determine if another method of treatment would have led to better patient outcomes. Furthermore, a one-year follow-up period may not be sufficient for drawing conclusions regarding functional outcomes after such fractures. We also utilized a single implant (PHILOS) when other proximal humeral locking plates also exist.

## Conclusions

Our results suggest that the PHILOS plate is an effective system in providing fracture stabilization in three- and four-part proximal humeral fractures until union occurs. The usage of the PHILOS plate also allows early mobilization and a good functional outcome for patients. It is vital for the surgeon to be aware of the potential complications including but not limited to avascular necrosis of the humeral head, mal-union, and intra-articular screw perforation. Future work could involve comparing outcomes between different types of proximal humeral locking plates.
